# Meningococcal carriage in Norwegian teenagers: strain characterisation and assessment of risk factors

**DOI:** 10.1017/S0950268820000734

**Published:** 2020-03-31

**Authors:** S. V. Watle, D. A. Caugant, G. Tunheim, T. Bekkevold, I. Laake, O. B. Brynildsrud, L. M. Næss

**Affiliations:** 1 Division of Infection Control and Environmental Health, Norwegian Institute of Public Health, P.O. Box 222 Skøyen, 0213 Oslo, Norway; 2 Faculty of Medicine, Institute of Health and Society, University of Oslo, P.O. Box 1078 Blindern, 0316 Oslo, Norway

**Keywords:** Carriage, meningococcal vaccines, *Neisseria meningitidis*, smokeless tobacco, Swedish snus, teenager, whole genome sequencing

## Abstract

Teenagers have a higher risk of invasive meningococcal disease (IMD) than the general population. This cross-sectional study aimed to characterise strains of *Neisseria meningitidis* circulating among Norwegian teenagers and to assess risk factors for meningococcal carriage. Oropharyngeal swabs were collected from secondary-school students in southeastern Norway in 2018–2019. Meningococcal isolates were characterised using whole genome sequencing. Risk factors for meningococcal carriage were assessed from questionnaire data. Samples were obtained from 2296 12–24-year-olds (majority 13–19-year-olds). *N. meningitidis* was identified in 167 (7.3%) individuals. The highest carriage rate was found among 18-year-olds (16.4%). Most carriage isolates were capsule null (40.1%) or genogroup Y (33.5%). Clonal complexes cc23 (35.9%) and cc198 (32.3%) dominated and 38.9% of carriage strains were similar to invasive strains currently causing IMD in Norway. Use of Swedish snus (smokeless tobacco) (OR 1.56, 95% CI 1.07–2.27), kissing >two persons/month (OR 2.76, 95% CI 1.49–5.10) and partying >10 times/3months (OR 3.50, 95% CI 1.45–8.48) were associated with carriage, while age, cigarette smoking, sharing of drinking bottles and meningococcal vaccination were not. The high meningococcal carriage rate among 18-year-olds is probably due to risk-related behaviour. Use of Swedish snus is possibly a new risk factor for meningococcal carriage. Almost 40% of circulating carriage strains have invasive potential.

## Introduction

Invasive meningococcal disease (IMD) is caused by *Neisseria meningitidis*, the meningococcus. Despite improved treatment and the development of effective vaccines, mortality remains high even in high-income countries. Many surviving patients suffer sequelae such as skin scarring, limb amputation, hearing loss or learning disabilities [1]. Meningococci are classified into 12 serogroups based on the structure of the polysaccharide capsule. IMD is usually caused by serogroups A, B, C, W, X or Y, which all have epidemic potential. Uncapsulated meningococci seldom cause invasive disease, but are commonly detected in the oropharynx in healthy individuals [2].

Meningococcal carriage is a prerequisite for developing IMD and essential for transmission. *N. meningitidis* is only found in humans and is transmitted through respiratory secretions resulting in transient nasopharyngeal colonisation. Carriage is usually asymptomatic and peaks in adolescents and young adults [3]. Smoking, kissing, overcrowding and frequenting bars are known risk factors for meningococcal carriage [4]. Only a small fraction of carriers will develop invasive disease, usually shortly after acquisition of the bacterium.

The incidence of IMD in Norway has been below 0.5 per 100 000 in the general population in the last 3 years [5]. After a peak in 2010 with 5.3 cases per 100 000, the mean incidence in 15–19-year-olds was 1.2 per 100 000 in the past 3 years. Most cases in Norwegian teenagers have been associated with the month-long ‘russ celebration’, a tradition with heavy drinking and partying among graduates from upper secondary school [6]. Serogroup Y dominated (75%) in 15–19-year-olds, even though the two fatal cases in 2017–2019 were caused by serogroups C and W, clonal complex (cc) 11 [5]. In the same period, the mean incidence in other age groups was 0.8 per 100 000 in children <5 years and 0.3 per 100 000 in both 5–14-year-olds and adults >19 years. Serogroup B dominated in children <5 years, while cases in the 5–14-year-olds and adults >19 years were caused by serogroups B, C, W and Y.

Meningococcal vaccination is not part of the national immunisation programme in Norway. A substantial increase in the incidence of IMD was observed in teenagers involved in the russ celebration in 2009–2010. Therefore, the Norwegian Institute of Public Health (NIPH) started recommending meningococcal ACWY conjugate vaccine (MCV4) to 17–19-year-olds engaged in activities that increase the risk of IMD, such as smoking, sharing drinking bottles and participating in youth gatherings, in 2011. The recommendation was extended to 16–19-year-olds from 2012. The school health services usually administer vaccination at the student's own cost. Vaccination is usually offered during the last year of upper secondary school. Uptake of vaccination among graduating students aged 18–19 years has increased from 27% in 2015 to 51% in 2019 [7].

The aims of this study were to investigate the prevalence of meningococcal carriage in Norwegian teenagers, to characterise circulating carriage strains of *N. meningitidis* and to identify risk factors for meningococcal carriage in this age group. These data are needed to improve public health recommendations regarding IMD in teenagers and to evaluate if meningococcal vaccines should be implemented in the national immunisation programme.

## Materials and methods

### Study design and inclusion of participants

Students in lower (grades 8–10) and upper (grades 11–14) secondary schools in three counties in southeastern Norway were recruited in a cross-sectional study. Only schools with >300 students were invited to participate. The study was conducted in two sampling periods, October–November 2018 (county 1) and February–April 2019 (counties 2 and 3), due to laboratory capacity. Students and parents received information about the study through SMS or e-mail from the school administrations. Students aged ≥16 years consented on their own behalf, while parental consent was requested for younger students. The study was approved by the Regional Committee for Medical and Health Research Ethics, Southeast Norway (reference number 2018/465).

### Sampling and bacterial identification

Swabbing of the posterior pharyngeal wall and one tonsil was performed on school grounds using a sterile cotton swab (Copan Diagnostics, CA, USA). Samples were plated on site on chocolate agar (with lincomycin 2.0 mg, colistin sulphate 12.0 mg, amphotericin B 2.0 mg and trimethoprim lactate 13.0 mg). Plates were transported to the laboratory in styrofoam containers at room temperature and then incubated for 24–48 h at 35 °C with 5% CO_2_ within 6 h of collection. Single colonies suspected to represent *N. meningitidis* were harvested and species identification was confirmed with MALDI-TOF mass spectrometry (Bruker Daltonik GmbH, Bremen, Germany). Meningococcal isolates were stored at −80 °C in Greaves solution.

### Molecular characterisation of *N. meningitidis* isolates

DNA from the confirmed isolates was extracted using MagNA Pure 96 (Roche Life Science, Basel, Switzerland). Whole genome sequencing (WGS) was performed using the MiSeq platform (Illumina Inc., San Diego, CA, USA) as described previously [8]. Genogroups, multilocus sequence types, PorA and FetA types were identified using the PubMLST database [9]. Isolates with deletions or stop codons in the capsule locus were identified as non-groupable (NG). Those lacking the capsule operon were classified as capsule null (*cnl*).

### Phylogenetic analysis

The genomes of the meningococcal carriage isolates were compared to the genomes of all invasive isolates in Norway submitted to the National Reference laboratory between January 2018 and July 2019 (*n* = 35). Phylogenetic trees were created by using the Neighbour-Joining algorithm on the pairwise distances across the 1605 loci defined in the core genome multilocus sequence typing (cgMLST) scheme v 1.0 [10]. In this calculation, incomplete loci were disregarded for the purpose of pairwise distance calculation. For the dominating invasive ccs in the study period, cc11 and cc23, minimum spanning trees were created based on the aforementioned allelic distance profiles and visualised using GrapeTree [11]. For the evaluation of differences between closely related invasive-carrier clusters, we evaluated all allelic differences across the 3050 loci defined in PubMLST (wgMLST) at the time of analysis (November 2019). Closely related invasive-carrier clusters were defined as a difference of <20 genes.

### Assessment of risk factors for meningococcal carriage and definition of variables

The participants were asked to complete an electronic questionnaire that assessed smoking habits, exposure to passive smoking at home, use of Swedish snus (smokeless tobacco), intimate kissing, sharing of drinking bottles, attendance of youth gatherings and parties, participation in the russ celebration, recent throat infection, recent use of antibiotics, parental background, parental education and the number of persons in the household. Using the unique personal identification number assigned to all residents of Norway, data from each participant were linked to their records of meningococcal vaccination in The Norwegian Immunisation Registry, SYSVAK [7]. The participants were considered vaccinated if they had received a meningococcal vaccine more than 2 weeks and less than 5 years before sampling (i.e. the expected duration of protection for MCV4 [12]). Age was defined as age in years at the time of sampling. In Norway, the attended school grade is based on birth cohort and therefore corresponds well with age.

### Study population

**Fig. 1.**
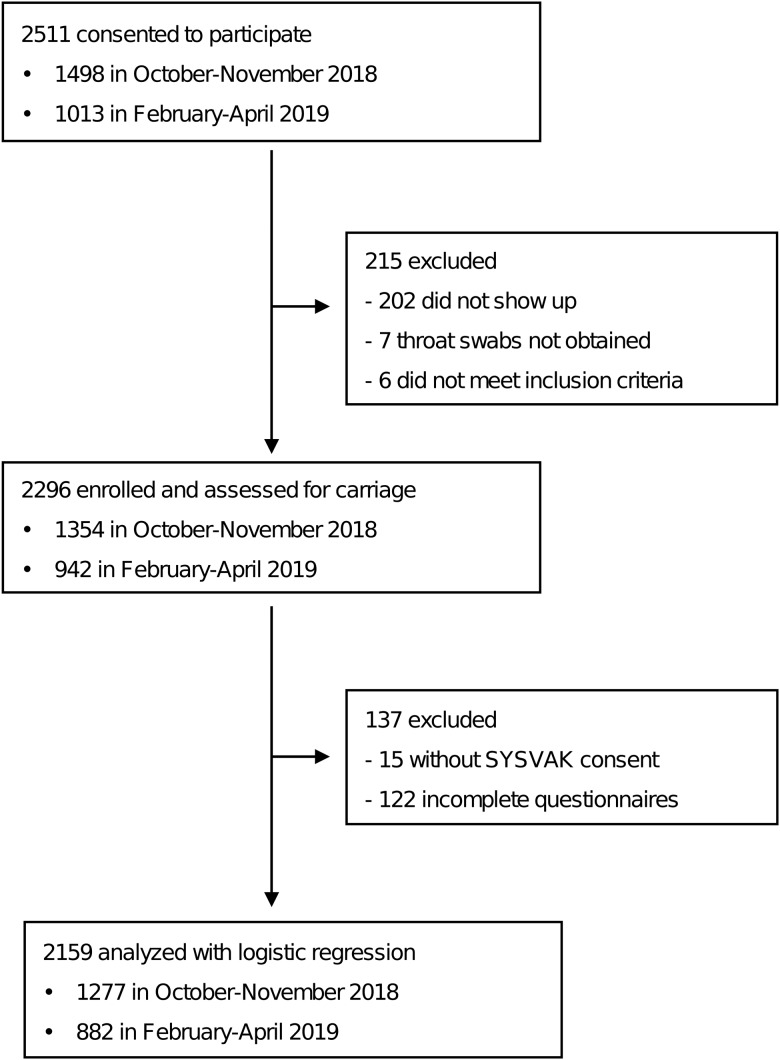
Flow-chart for inclusion of participants in the overall study with regards to sampling, consent for assessment of meningococcal vaccination status and completion of questionnaires. Number of participants in sampling period 1 (October–November 2018) and sampling period 2 (February–April 2019) are shown. SYSVAK = National Immunisation Registry SYSVAK.

Among the 106 schools invited to recruit students, 21 lower and 24 upper secondary schools participated in the study. In total, 2511 students consented and throat swabs were obtained from 2296 participants; 1354 students in October–November 2018 (59.0%) and 942 students in February–April 2019 (41.0%) (Fig. 1). The median number of students with throat swabs per school was 51 (range 10–80). All 2296 participants were included when assessing carriage prevalence. Among the 2296 participants from whom throat swabs were collected, 137 were excluded because of incomplete questionnaire data and/or unknown meningococcal vaccination status and 2159 (93.8%) individuals were included in the analyses of risk factors (Fig. 1).

### Statistical methods

We calculated carriage rates with 95% confidence intervals (CIs) based on cluster-robust standard error. When testing whether the carriage rate differed between sampling periods or genders, the Pearson *χ*^2^ test statistic was corrected with the second-order correction of Rao and Scott and converted into an *F* statistic [13]. The associations between potential risk factors and the risk of carriage were studied using a logistic model estimating odds ratios (ORs) and 95% CIs. The generalised estimating equations approach was used to fit the model in order to account for dependencies within schools. An exchangeable correlation structure was assumed. We used cluster-robust estimates of the standard errors. The multivariable model included smoking habits, exposure to passive smoking at home, use of Swedish snus, intimate kissing, sharing of drinking bottles, attendance of youth gatherings and parties, participation in the russ celebration, recent throat infection and parental background. For the variables assessing smoking habits and the use of snus, the response categories ‘daily’ and ‘occasionally’ were merged in the multivariable analysis. In addition, we adjusted for sampling period, gender, school grade (with grades 13 and 14 combined) and record of previous vaccination with MCV4. Use of antibiotics, parental education and number of persons in the household were not included in the multivariable analysis due to high degree of missing and/or inadequate/low quality of data. *P*-values <0.05 were considered statistically significant. All analyses were performed with Stata/SE 15.0 (Stata-Corp, College Station, Texas, USA).

## Results

### Characteristics of the participants

The median age of the 2296 participants from whom throat swabs were obtained was 16 years (range 12–24 years) and 68.5% attended upper secondary school. In lower secondary schools (grades 8–10), the majority of students (96.3%) were 13–15 years (range 12–16 years) whereas the majority of students (93.1%) in upper secondary schools (grades 11–14) were 16–18 years (range 15–24 years). Overall, 61.5% of participants were female.

### Carriage rate of *N. meningitidis*

**Fig. 2.**
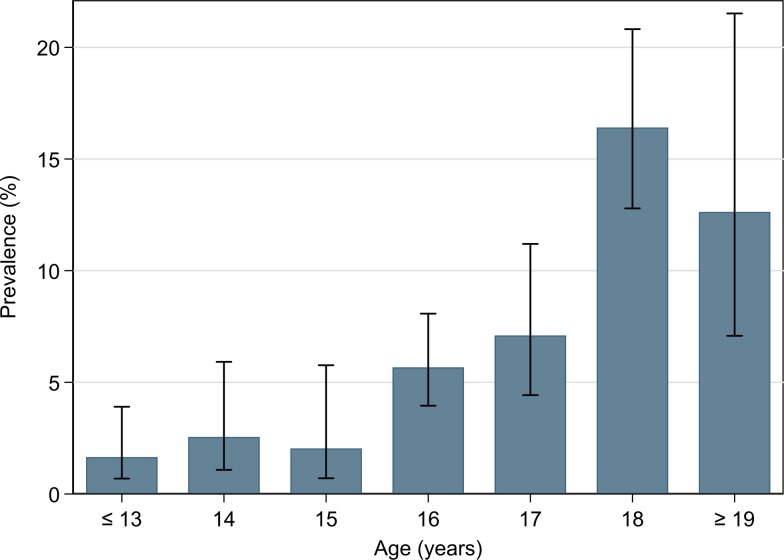
Prevalence of carriers of *N. meningitidis* by age (*n* = 2296; carriers of *N. meningitidis n* = 167). Error bars indicate 95% confidence intervals.

In total, 167 of the 2296 participants were identified as carriers of *N. meningitidis*. The overall carriage rate was 7.3% (95% CI 5.5–9.6%). There was no significant difference in carriage rate between the first and second sampling periods (7.8% (95% CI 5.3–11.2) *vs.* 6.6% (95% CI 4.3–9.8), *P* = 0.556), or between boys and girls (7.8% (95% CI 5.1–11.6) *vs.* 6.9% (95% CI 5.2–9.2), *P* = 0.540). The majority of the meningococcal isolates (91.6%) were found in students attending upper secondary schools. The carriage rate in lower secondary school was 1.9% (95%CI 0.9–3.9) and in upper secondary school 9.7% (95% CI 7.5–12.6). The highest carriage rate was found among the 18-year-olds (16.4% (95% CI 12.7–21.0%)) (Fig. 2).

### Molecular characterisation of carriage isolates

**Fig. 3.**
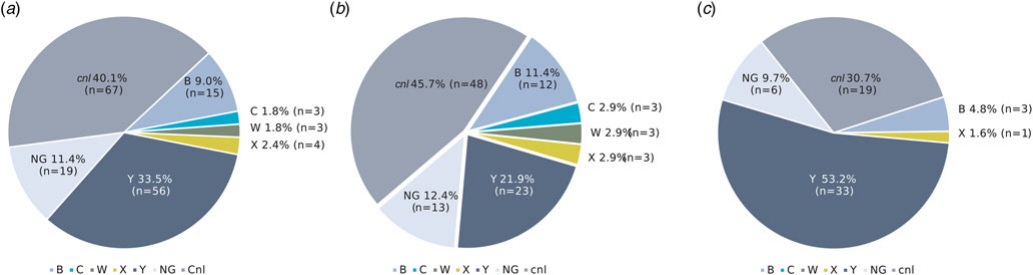
Genogroup distribution of carriage isolates of *N. meningitidis* in the study population in (a) overall (b) October–November 2018 and (c) February–April 2019. NG, non-groupable; cnl, capsule null locus.

Among the 167 carriage isolates, *cnl* dominated (40.1%), followed by genogroups Y (33.5%), NG (11.4%), B (9.0%), X (2.4%), C (1.8%) and W (1.8%) (Fig. 3a). In October–November 2018, *cnl* isolates dominated (45.7%) followed by genogroup Y (21.9%), whereas in February-April 2019 genogroup Y dominated (53.2%) followed by *cnl* (30.7%) (Fig. 3b and 3c). The distributions of genogroup Y and *cnl* between sampling periods were not significantly different (*P* = 0.06 and 0.12 for genogroup Y and *cnl*, respectively).

Among the 167 carriage isolates, 157 were assigned to 17 previously defined ccs (Table 1). Ten isolates representing six sequence types (STs) were unassigned to a cc. The most common ccs were cc23 (35.9%) and cc198 (32.3%). There were 28 previously defined STs and 11 new STs represented among the 167 isolates; ST-23 (33.5%) and ST-823 (26.4%) dominated. The majority of cc23 were ST-23 genogroup Y (86.7%) and the majority of cc198 were ST-823 *cnl* (81.5%). These two clones were found in 22 of 45 and 21 of 45 schools, respectively. Genogroups B, C, W and X isolates belonged to multiple STs. Among the 167 isolates, there were 48 PorA types (nine new variants), with P1.5-2, 10-1 (29.3%) and P1.18, 25–44 (22.2%) dominating and 20 FetA types (three new variants) with predominance of F4-1 (34.1%) and F1-43 (18.6%). The *fetA* gene was missing in 14 isolates (8.4%).

**Table 1.** Molecular characteristics of the *N. meningitidis* carriage isolates (*n* = 167)

cc	ST	Genogroup	PorA	FetA	No. of isolates
11	11	C	5, 2	F3-3	3
11	11	W	5, 2	F1-1	1
22	1061	W	18-1, 3	F1-22	1
22	1281	W	18-1, 3	F5-5	1
23	23	Y	5-1, 10-1	F4-1	6
23	23	Y	5-2, 10-1	F4-1	39
23	23	Y	5-2, 10-1	F5-8	1
23	23	Y	5-2, 10-1	F5-12	3
23	23	Y	5-2, 10-15	F5-12	1
23	23	Y	5-2, 10-15	F4-1	1
23	23	Y	5-2, 10-29	F4-1	1
23	23	NG	5-2, 10-1	F4-1	4
23	1655	Y	5-1, 10-1	F4-1	2
23	14 456^a^	Y	5-2, 10-1	F4-1	2
32	32	B	7-2, 16-134	F3-3	3
32	14 474^a^	NG	7-11, 16-29	F3-3	1
35	2906	NG	22-1, 14	–	1
41/44	41	*cnl*	7-2, 4-41^a^	F1-5	1
41/44	41	B	7-20, 4	F1-2	1
41/44	43	B	7-4, 1	F1-5	1
41/44	2578	*cnl*	18, 25-1	F1-5	1
41/44	14 238^a^	B	19-1, 15-1	F4-17	2
41/44	14 478^a^	B	19-1, 26-10^a^	F1-7	1
53	53	*cnl*	7, 30-4	F1-2	1
53	53	*cnl*	7-2, 30	F1-2	1
53	14 237^a^	*cnl*	7-2, 30	F1-2	1
53	14 477^a^	*cnl*	7, 30-3	F1-2	1
162	162	B	7-2, 4-30^a^	F5-9	2
162	162	B	7-2, 4	F5-9	1
175	175	NG	25-11, 15-25	F5-1	2
175	175	NG	22-11, 15-75^a^	–	1
178	178	NG	19-5, 15-23	F1-7	4
192	192	*cnl*	18-11, 42-1	–	1
192	14 457^a^	*cnl*	18-11, 42-2	–	1
198	198	*cnl*	18, 25-1	F5-5	1
198	823	*cnl*	5-2, 25-44	F4-1	1
198	823	*cnl*	18, 25-14	–	4
198	823	*cnl*	18, 25-15	F5-5	1
198	823	*cnl*	18, 25-37	F5-5	7
198	823	*cnl*	18, 25-44	–	3
198	823	*Cnl*	18, 25-44*	F1-43	24
198	823	*cnl*	18, 25-44	F5-5	2
198	823	*cnl*	18-23, 25-37	F5-5	1
198	823	*cnl*	18-48, 25-44	F1-43	1
198	2384	*cnl*	18, 25-44	F1-43	4
198	2384	*cnl*	18, 25-44	–	1
198	14 235^a^	*cnl*	18, 25-44	F1-43	1
198	14 236^a^	*cnl*	18, 25-44	F1-43	1
198	14 236^a^	*cnl*	18, 25-44	–	1
198	14 453^a^	*cnl*	18, 25-37	F5-5	1
269	269	NG	17-1, 23	F1-7	1
865	865	B	7-1, 1	F1-6	1
865	865	B	7-50, 1-10	F1-6	3
1117	1117	*cnl*	18-1, 30	F3-7	1
1136	1136	*cnl*	18-4, 25	F2-1	1
1136	1136	*cnl*	18-4, 25-6	F4-1	1
1157	1157	NG	17^a^ –	F5-36	1
u.a.	5063	X	5-1, 2-2	F5-179^a^	2
u.a.	5063	X	5-1, 2-92^a^	F5-179^a^	1
u.a.	5063	X	5-1, 2-88	–	1
u.a.	6798	NG	5-1, 10-26	F5-5	1
u.a.	7129	*cnl*	12-6, 13-22	F5-5	1
u.a.	10 866	NG	19-1, 14-13	F5-7	1
u.a.	13 041	*cnl*	12-1, 16-173	F5-5	1
u.a.	14 234^a^	NG	22, 14-13	F5-7	2

cc, clonal complex; ST, sequence type; PorA, porin A; FetA, ferric enterobactin receptor; NG, non-groupable; *cnl*, capsule null locus; u.a., unassigned; –, gene not present.

a New variants.

**Fig. 4.**
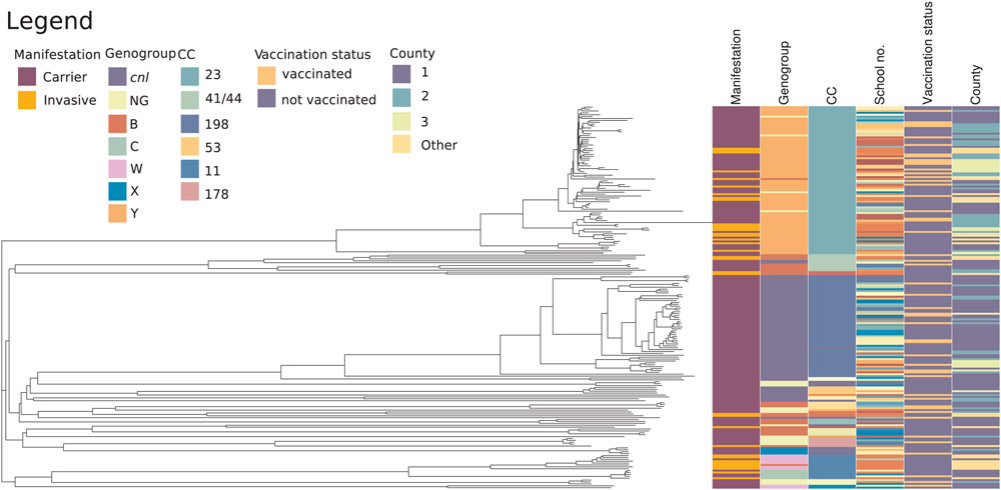
Phylogenetic relationships of the 167 *N. meningitidis* carriage isolates together with 35 invasive isolates from cases occurring in Norway from January 2018 to July 2019. Clinical manifestation, genogroup and clonal complex of the isolates, school number, vaccination status and county of the origin of the carriers and patients are displayed on the right. The color codes are shown in the figure. Color codes for school numbers are not listed in the legend since invasive isolates did not have school numbers. cc, clonal complex; cnl, capsule null locus; NG, non-groupable.

**Fig. 5.**
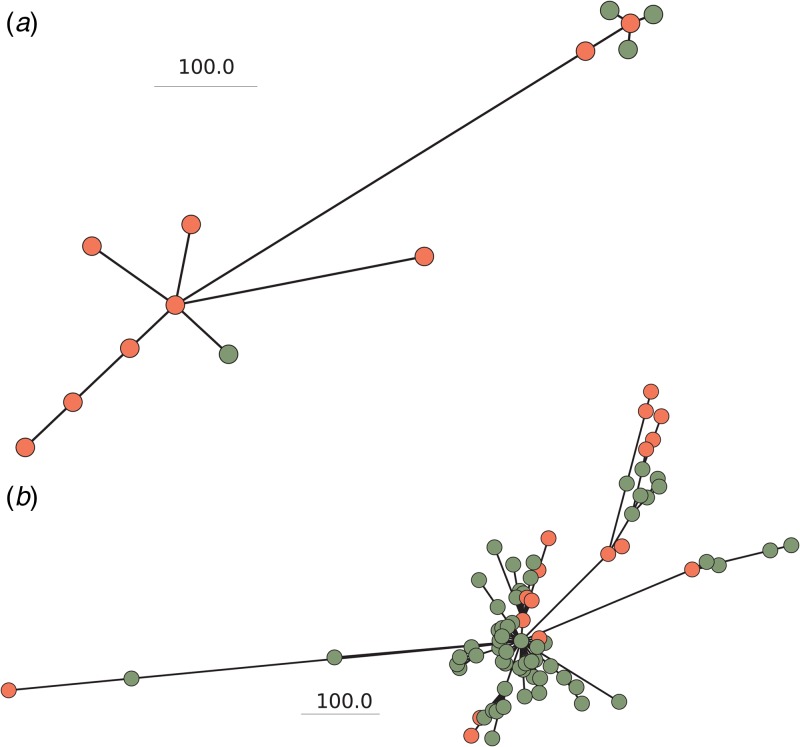
Grape trees of carrier (green) and invasive (red) isolates of *N. meningitidi*s for (a) cc11 (*n* = 4 carriage isolates; *n* = 7 invasive isolates) and (b) cc23 (*n* = 60 carriage isolates; *n* = 17 invasive isolates). In A, upper right corner represents genogroup C and lower left corner genogroup W. cc, clonal complex.

A phylogenetic tree was generated to illustrate the relationships among the 167 carrier isolates and 35 recent invasive Norwegian isolates (Fig. 4). Of the carriage isolates, 38.9% were both capsulated and belonged to ccs presently causing invasive disease in Norway. Isolates of cc23 had a median allelic distance of 120.0 (range 4.0–732.0, s.d. 142.2) and cc198 isolates a median distance of 149.0 (range 3.0–827.0, s.d. 169.6), showing that the latter group was slightly more diverse. There was no apparent association between cc and school number (*P* = 0.49), county (*P* = 0.31) or vaccination status (*P* = 0.49) for the carriage isolates, as evaluated using the *χ*^2^-test. The three C:P1.5,2:F3-3:ST-11 carriage isolates were very closely related to the invasive isolate with the same composition, differing in less than 20 genes in wgMLST (Fig. 5, panel a, upper right corner). The cc23 carriage and invasive isolates appeared more genetically distant (Fig. 5, panel b), although six carriage isolates also differed by less than 20 genes from an invasive isolate.

### Risk factors associated with carriage of *N. meningitidis*

Of the 2159 participants assessed for risk factors, 157 were carriers of *N. meningitidis*. In general, upper secondary school students reported higher exposure to risk factors than lower secondary school students (Table 2). Occasional cigarette smoking was reported by 18.4% of the upper secondary school students, but only 2.3% of lower secondary school students. Daily smoking was rare in both upper and lower secondary schools students (⩽0.5%). In upper secondary school student, 11.8% used Swedish snus daily and 9.3% occasionally, while in lower secondary school students, 0.6% used Swedish snus daily and 1.3% occasionally. Less than 5% of participants had used e-cigarettes (vaping) or waterpipe, of which few were daily users (⩽0.6% and ⩽0.1%, respectively). While 47.1% of upper secondary school students participated in the russ celebration, only 1.2% of lower secondary school students did. Vaccination with MCV4 was registered for 0.6% and 25.1% of lower and upper secondary school students, respectively.

**Table 2.** Characteristics of the population assessed for carriage of *N. meningitidis* (*n* = 2159)

Variable	Response	No. of participants in all schools (%) (*n* = 2159)	No. of participants in lower secondary school (%) (*n* = 691)	No. of participants in upper secondary school (%) (*n* = 1468)
Sampling period	October–November 2018	1277 (59.1)	412 (59.6)	865 (58.9)
	February–April 2019	882 (40.9)	279 (40.4)	603 (41.1)
Gender	Male	819 (37.9)	324 (46.9)	495 (33.7)
	Female	1340 (62.1)	367 (53.1)	973 (66.3)
Grade (median age in years)	8 (13)	–	244 (35.3)	–
	9 (14)	–	229 (33.1)	–
	10 (15)	–	218 (31.5)	–
	11 (16)	–	–	513 (34.9)
	12 (17)	–	–	445 (30.3)
	13 (18)	–	–	497 (33.9)
	14^a^ (21)	–	–	13 (0.9)
Cigarette smoking	No	1864 (86.3)	674 (97.5)	1190 (81.1)
	Occasionally	286 (13.2)	16 (2.3)	270 (18.4)
	Daily	9 (0.4)	1 (0.1)	8 (0.5)
Exposure to passive smoking at home	No	1784 (82.6)	614 (88.9)	1170 (79.7)
	Yes	375 (17.4)	77 (11.1)	298 (20.3)
E-cigarette smoking	No	2075 (96.1)	681 (98.6)	1394 (95.0)
	Occasionally	75 (3.5)	10 (1.5)	65 (4.4)
	Daily	9 (0.4)	0 (0.0)	9 (0.6)
Use of waterpipe	No	2108 (97.6)	690 (99.9)	1418 (96.6)
	Occasionally	50 (2.3)	1 (0.1)	49 (3.3)
	Daily	1 (0.0)	0 (0.0)	1 (0.1)
Use of Swedish snus	No	1836 (85.0)	678 (98.1)	1158 (78.9)
	Occasionally	146 (6.8)	9 (1.3)	137 (9.3)
	Daily	177 (8.2)	4 (0.6)	173 (11.8)
No. of persons kissed the last month (intimate kissing)	None	1140 (52.8)	576 (83.4)	564 (38.4)
	1	626 (29.0)	81 (11.7)	545 (37.1)
	2	164 (7.6)	18 (2.6)	146 (9.9)
	> 2	229 (10.6)	16 (2.3)	213 (14.5)
Frequency of sharing drinking bottles	None	611 (28.3)	295 (42.7)	316 (21.5)
	Monthly	546 (25.3)	163 (23.6)	383 (26.1)
	Weekly	768 (35.6)	185 (26.8)	583 (39.7)
	Daily	234 (10.8)	48 (6.9)	186 (12.7)
No. of times attending a party or youth gathering the previous three months	None	639 (29.6)	339 (49.1)	300 (20.4)
	1-3 times	806 (37.3)	291 (42.1)	515 (35.1)
	4-6 times	337 (15.6)	44 (6.4)	293 (20.0)
	7-10 times	194 (9.0)	13 (1.9)	181 (12.3)
	> 10 times	183 (8.5)	4 (0.6)	179 (12.2)
Participation in russ celebration	No	1459 (67.6)	683 (98.8)	776 (52.9)
	Yes	700 (32.4)	8 (1.2)	692 (47.1)
Throat pain or upper respiratory infection previous week	No	1321 (61.2)	463 (67.0)	858 (58.4)
	Yes	838 (38.8)	228 (33.0)	610 (41.6)
Use of antibiotics previous 2 weeks^b^	No	1932 (89.4)	634 (91.8)	1298 (88.4)
	Yes	129 (6.0)	27 (3.9)	102 (6.9)
	No data	98 (4.5)	30 (4.3)	68 (4.6)
Parental background	Norwegian	1659 (76.8)	559 (80.1)	1100 (74.9)
	Other	500 (23.2)	132 (19.1)	368 (25.1)
Parental education^b^	Unknown	188 (8.7)	83 (12.0)	105 (7.2)
	Primary education	38 (1.8)	8 (1.2)	30 (2.0)
	Secondary education	369 (17.1)	86 (12.4)	283 (19.3)
	Tertiary education	1564 (72.4)	514 (23.8)	1050 (71.5)
No. of persons in the household^b^	0	11 (0.5)	0 (0.0)	11 (0.7)
	1	157 (7.3)	33 (47.8)	124 (8.4)
	2	463 (21.4)	118 (17.1)	345 (23.5)
	3	826 (38.3)	317 (45.9)	509 (34.7)
	4	478 (22.1)	160 (23.2)	318 (21.7)
	>4	215 (10.0)	59 (8.5)	156 (10.6)
	No data	9 (0.4)	4 (0.6)	5 (0.3)
Vaccinated with ACWY conjugate vaccine	No	1786 (82.7)	687 (99.4)	1099 (74.9)
	Yes	373 (17.3)	4 (0.6)	369 (25.1)

a Grade 14 comprises students with vocational education taking supplementary studies to qualify for higher education

b Variables not included in multivariable analysis

Both use of Swedish snus (OR 1.56, 95% CI 1.07–2.27) and participation in the russ celebration (OR 2.85, 95% CI 1.62–5.02) were associated with a higher risk of carriage of *N. meningitidis* (Table 3). We also observed a positive association between carriage and the numbers of persons kissed as well as the number of times attending parties or youth gatherings. Compared to not having kissed, ORs for number of persons kissed the last month were 2.73 (95% CI 1.58–4.70) for one person, 3.03 (95% CI 1.44–6.36) for two persons and 2.76 (95% CI 1.49–5.10) for more than two persons. Compared to not having attended parties or youth gatherings, ORs for attendance the last three months were 2.14 (95% CI 1.01–4.54) for 1–3 times, 2.20 (95% CI 1.03–4.71) for 4–6 times, 2.84 (95% CI 1.27–6.36) for 7–10 times and 3.50 (95% CI 1.45–8.48) for >10 times. There was no significant association between carriage and sampling period, gender, throat pain or upper respiratory infection, active or passive cigarette smoking, use of waterpipe or e-cigarettes, sharing drinking bottles or parental background. Carriage rate increased with school grade (hence age) in the univariate analysis, but not when adjusting for other risk factors. Previous vaccination with MCV4 was not associated with carriage, neither carriage of all genogroups (Table 3) nor of vaccine-specific genogroups (OR 1.00; 95% CI 0.53–1.88).

**Table 3.** Univariate and multivariable analysis of risk factors for meningococcal carriage (*n* = 2159; carriers of *N. meningitidis* = 157)

Variable	Response	No. of carriers (%)	Univariate analysis OR (95% CI)	*P*-value	Multivariable analysis OR (95% CI)	*P*-value*
Sampling period	October–November 2018	100 (7.8)	1 (Ref)		1 (Ref)	
	February–April 2019	57 (6.5)	0.78 (0.42–1.46)	0.444	0.90 (0.59–1.36)	0.610
Gender	Male	64 (7.8)	1 (Ref)		1 (Ref)	
	Female	93 (6.9)	0.84 (0.57–1.25)	0.392	0.79 (0.53–1.17)	0.235
Grade (median age in years)	8 (13)	4 (1.6)	1 (Ref)		1 (Ref)	
	9 (14)	6 (2.6)	1.49 (0.63–3.54)	0.369	1.15 (0.46–2.90)	0.764
	10 (15)	4 (1.8)	1.05 (0.31–3.59)	0.937	0.57 (0.17–1.96)	0.372
	11 (16)	31 (6.0)	3.37 (1.34–8.47)	0.010*	0.87 (0.31–2.42)	0.793
	12 (17)	30 (6.7)	3.84 (1.49–9.92)	0.005*	0.64 (0.20–2.07)	0.456
	13-14^a^ (18)	82 (16.1)	9.99 (4.10–24.33)	< 0.001*	0.96 (0.31–3.02)	0.950
Cigarette smoking	No	100 (5.4)	1 (Ref)		1 (Ref)	
	Yes	57 (19.3)	3.43 (2.33–5.05)	< 0.001*	1.35 (0.92–2.00)	0.126
Exposure to passive smoking at home	No	125 (7.0)	1 (Ref)		1 (Ref)	
	Yes	32 (8.5)	1.29 (0.88–1.89)	0.193	1.04 (0.67–1.62)	0.865
E-cigarette smoking	No	141 (6.8)	1 (Ref)		1 (Ref)	
	Yes	16 (19.1)	3.11 (1.79–5.39)	< 0.001*	1.63 (0.92–2.88)	0.092
Use of waterpipe	No	146 (6.9)	1 (Ref)		1 (Ref)	
	Yes	11 (21.6)	3.60 (2.06–6.30)	< 0.001*	1.60 (0.83–3.10)	0.161
Use of Swedish snus	No	93 (5.1)	1 (Ref)		1 (Ref)	
	Yes	64 (19.8)	3.93 (2.49–6.19)	< 0.001*	1.56 (1.07–2.27)	0.020*
No. of persons kissed the last month (intimate kissing)	None	24 (2.1)	1 (Ref)		1 (Ref)	
	1	68 (10.9)	5.03 (2.91–8.73)	< 0.001*	2.73 (1.58–4.70)	< 0.001*
	2	26 (15.9)	7.28 (3.34–15.84)	< 0.001*	3.03 (1.44–6.36)	0.001*
	> 2	39 (17.0)	7.85 (4.61–13.38)	< 0.001*	2.76 (1.49–5.10)	0.002*
Frequency of sharing drinking bottles	None	17 (2.8)	1 (Ref)		1 (Ref)	
	Monthly	43 (7.9)	2.39 (1.33–4.30)	0.003*	1.39 (0.69–2.79)	0.351
	Weekly	77 (10.0)	2.88 (1.69–4.93)	< 0.001*	1.36 (0.71–2.62)	0.359
	Daily	20 (8.6)	2.45 (1.32–4.54)	0.005*	1.30 (0.65–2.60)	0.463
No. of times attending a party or youth gathering the previous 3 months	None	8 (1.3)	1 (Ref)		1 (Ref)	
	1–3 times	41 (5.1)	3.91 (1.81–8.40)	< 0.001*	2.14 (1.01–4.54)	0.048*
	4–6 times	33 (9.8)	7.53 (3.65–15.55)	< 0.001*	2.20 (1.03–4.71)	0.041*
	7–10 times	32 (16.5)	13.61 (6.22–29.75)	< 0.001*	2.84 (1.27–6.36)	0.011*
	>10 times	43 (23.5)	21.12 (9.67–46.14)	< 0.001*	3.50 (1.45–8.48)	0.005*
Participation in russ celebration	No	40 (2.7)	1 (Ref)		1 (Ref)	
	Yes	117 (16.7)	6.43 (4.12–10.02)	< 0.001*	2.85 (1.62–5.02)	< 0.001*
Throat pain or upper respiratory infection previous week	No	88 (6.7)	1 (Ref)		1 (Ref)	
	Yes	69 (8.2)	1.18 (0.79–1.77)	0.412	0.88 (0.61–1.26)	0.486
Vaccinated with ACWY conjugate vaccine, carriage of all genogroups	No	109 (6.1)	1 (Ref)		1 (Ref)	
	Yes	48 (12.9)	2.38 (1.53–3.69)	< 0.001*	0.86 (0.59–1.26)	0.442

OR, odds ratio; CI, confidence interval.

a Grade 14 comprises students with vocational education taking supplementary studies to qualify for higher education.

* *P* values <0.05 are considered statistically significant.

## Discussion

The overall carriage rate of *N. meningitidis* was 7.3%. Meningococcal carriage was associated with the use of Swedish snus, intimate kissing, attending parties or youth gatherings and participation in the russ celebration. Age, cigarette smoking and MCV4 vaccination was not associated with carriage. Isolates belonging to *cnl* cc198 and genogroup Y cc23 dominated. Almost 40% of the carriage isolates were similar to invasive isolates currently causing IMD in Norway.

The main strengths of this study were that over 90% of the throat swabs were collected by one study team member, plated on site and analysed at the meningococcal reference laboratory, which probably increased the quality of the samples. The study also had potential limitations. A higher proportion of upper secondary school students were included since participants over 16 years age, not needing parental consent, could be recruited on site. This may have overestimated carriage rate in the overall study population. Assessment of risk factors might have been affected by information bias as students who completed questionnaires in the company of parents or peers could have been influenced to either under- or over-report risk-behaviour.

Meningococcal carriage has decreased in Norway in the last 30 years, from 28.3% in 15–24-year-olds in 1991 [14] to 7.3% in the present study. Studies in Europe have shown variable carriage rates in the past decade. Rates below 10% was found in an ongoing study in Swedish university students [15], in Italy in 14–19-year-olds 2012–2013 [16] and in 14–21-year-olds in 2016 [17] and in Turkey in 10–24-year-olds in 2015 [18]. Higher carriage rates than in our study have been observed in the UK in 18–19-year-olds in 2015–2016 (14–46%) [19] and in the Netherlands in 13–23-year-olds in 2013–2014 (16%) [20]. The latter two studies included university students, which could explain the higher carriage rates. Despite increasing carriage prevalence with age in our study, age was not significantly associated with carriage when adjusting for other risk factors. Risk-behaviour rather than age influenced the risk of carriage, in concordance with the Dutch study [20].

To our knowledge, our study is the first to report the use of Swedish snus as a possible risk factor for meningococcal carriage. Swedish snus is a form of moist and ground smokeless tobacco put under the upper lip either in loose powder or portioned in small cellulose pouches. Swedish snus is prohibited in all EU/EEA countries except Sweden and Norway. However, Swedish snus is also marketed in the USA and 80% of American 12–17-year-olds reported to have tried snus pouches in 2013–2015 [21]. Use of Swedish snus has increased over the last 20 years in Norway and Sweden, and 25% of 16–24-year-olds in Norway used Swedish snus daily or occasionally in 2018 [22]. This corresponds to data from 16 to 24-year-olds in our study. Nicotine plasma levels have been reported to be higher after using Swedish snus compared to cigarettes and nicotine gum [23]. Nicotine is believed to have immunomodulatory effects and may suppress antibody production [24]. Moreover, nicotine increases biofilm formation of common oral bacteria [25]. Both capsulated and uncapsulated strains of *N. meningitidis* have shown to produce biofilm in human bronchial epithelial cells [26]. A combination of more optimal conditions for bacterial colonisation through induction of biofilm and a weaker mucosal immune defence, might explain the increased risk of meningococcal carriage in people using Swedish snus.

Participation in the russ celebration increased the risk of carriage almost threefold in our study. Bacterial transmission is probably high during this event through overcrowding, sharing of bottles, intimate kissing and excessive alcohol consumption [6]. IMD cases are annually linked to the russ celebration in Norway. Similarly, first year university students in the UK and USA, with similar risk behaviour as in the russ celebration, also have a higher risk of meningococcal carriage and IMD [4, 27]. High alcohol consumption has been associated with carriage [28]. This relationship was not assessed in our study, but needs further evaluation.

A relationship between cigarette smoking and carriage has previously been reported in Norway [14], in a carriage study in the UK from 1999 [4], as well as in a recent study in South Australia from 2019 [29]. In contrast, such a relationship was not found in the present study, or in university students in USA in 1992–1993 [28]. The studies from Norway and the UK were conducted in the 1990s, before the European Council recommended comprehensive smoke-free laws in EU member states and when smoking rates were high [30]. The low level of smoking in our study could explain the lack of an association between carriage and smoking.

Along with an increasing uptake of MCV4 among the graduating students involved in the russ celebration, the incidence of IMD in this group has decreased in the last decade. However, vaccination with MCV4 did not have an impact on carriage in our study. The effects of MCV4 on carriage and herd immunity are uncertain [31] and needs further assessment.

The dominant clones among the 167 isolates of *N. meningitidis* were observed in many of the schools and in all three counties. The majority of the isolates were *cnl* or NG, in accordance with recent studies in adolescents and young adults in Europe [15, 16, 19, 20, 32]. In our study, c*nl* cc198 dominated, as recently reported in a study among Italian teenagers [16] and in the ongoing study in Swedish university students [15]. The lack of capsular expression is thought to increase the meningococcus' ability to colonise the nasopharyngeal mucosa [33]. C*nl* strains are usually apathogenic since the meningococcal capsule is a major virulence factor [34]. In rare occasions, however, *cnl* cc198 strains have caused IMD in immunocompetent individuals [35–37].

Genogroup Y cc23 dominated among the isolates expressing capsular genes. Similar findings were reported in a previous carriage study in Norway among 13–14-year-olds in 1989 [38] and from the ongoing carriage study in Swedish university students [15]. Carriage of genogroup Y has been rare in adolescents and young adults in other European countries the last years [16, 18–20]. IMD caused by genogroup Y has increased in several European countries in the last decades [39–42]. This genogroup previously caused IMD mainly in adults [40], but it has also affected adolescents and young adults the last decade [41, 43]. In 2018, 50% of the overall IMD cases in Norway and five of seven cases in teenagers were caused by genogroup Y cc23.

Whereas genogroup B has dominated among isolates expressing capsular genes in carriage studies among adolescents and young adults in Europe the last 10 years [19, 20, 44], only 10% of the carriage isolates in our study were genogroup B. Correspondingly, there has not been any genogroup B IMD cases in teenagers in Norway in the previous 5 years. The relatively high circulation of serogroup B in Norway in the late 1990s [45] might have led to a higher level of natural immunity in the population, which may explain our findings.

We found four ST-11 carriage isolates in our study. Analyses of the three C:P1.5,2:F3-3:ST-11 isolates using cgMLST revealed a close similarity to a Norwegian invasive isolate from the same time period in a patient residing in the same county. After an increase in IMD caused by genogroup C ST-11 in Europe in the late 1990s, implementation of meningococcal C conjugate vaccines in many immunisation programmes across Europe have been effective in reducing the incidence [46]. Genogroup C IMD has been rare in Norway in the last 5 years, even though meningococcal vaccines are not included in the national immunisation programme.

Only one of the three genogroup W isolates represented the hypervirulent clone W:P1.5,2:F1-1:ST11. This clone has been seen among Norwegian invasive isolates the last years. A long-term increase such as seen in West Africa [47] and South America [48] in the early 2000s and in some European countries in recent years [49] has not been observed in Norway.

Genogroup X isolates, which has also been reported among teenagers in other European countries [16, 20, 32, 44], were found in three individuals in our study. IMD caused by genogroup X is uncommon in Western Europe [46], but an increase has been observed in Eastern Europe recently [50]. Differences between countries and fluctuations in genogroup distribution may be explained by emergence of hypervirulent clones, natural immunity, exposure to different environments and behavioural risk factors, as well as variations in vaccination policy.

## Conclusion

Carriage of *N. meningitidis* was 7.3% with a peak of 16.4% in 18-year-olds and is probably due to age-related risk behaviour, supporting current national recommendations regarding meningococcal vaccination of teenagers. The finding that Swedish snus might be a new risk factor for meningococcal carriage needs further investigation. Even though most of the circulating isolates of *N. meningitidis* lacked capsule-expressing genes suggesting a low risk for development of IMD, more than one-third of the circulating isolates have invasive potential. Analyses of the degree of natural protection as well as health economic evaluations are necessary to evaluate the demand for a meningococcal vaccination programme for teenagers in Norway.
